# Multifocal microscopy for functional imaging of neural systems

**DOI:** 10.1117/1.NPh.11.S1.S11515

**Published:** 2024-09-17

**Authors:** Nizan Meitav, Inbar Brosh, Limor Freifeld, Shy Shoham

**Affiliations:** a Technion - Israel Institute of Technology, Department of Biomedical Engineering, Kiryat HaTechnion, Haifa, Israel; b NYU Grossman School of Medicine, Tech4Health Institute and Departments of Neuroscience and Ophthalmology, New York, New York, United States

**Keywords:** multifocal grating, multifocal microscopy, volumetric imaging, fluorescence microscopy, light-field microscopy

## Abstract

**Significance:**

Rapid acquisition of large imaging volumes with microscopic resolution is an essential unmet need in biological research, especially for monitoring rapid dynamical processes such as fast activity in distributed neural systems.

**Aim:**

We present a multifocal strategy for fast, volumetric, diffraction-limited resolution imaging over relatively large and scalable fields of view (FOV) using single-camera exposures.

**Approach:**

Our multifocal microscopy approach leverages diffraction to image multiple focal depths simultaneously. It is based on a custom-designed diffractive optical element suited to low magnification and large FOV applications and customized prisms for chromatic correction, allowing for wide bandwidth fluorescence imaging. We integrate this system within a conventional microscope and demonstrate that our design can be used flexibly with a variety of magnification/numerical aperture (NA) objectives.

**Results:**

We first experimentally and numerically validate this system for large FOV microscope imaging (three orders-of-magnitude larger volumes than previously shown) at resolutions compatible with cellular imaging. We then demonstrate the utility of this approach by visualizing high resolution three-dimensional (3D) distributed neural network at volume rates up to 100 Hz. These demonstrations use genetically encoded ${\mathrm{Ca}}^{2+}$ indicators to measure functional neural imaging both *in vitro* and *in vivo.* Finally, we explore its potential in other important applications, including blood flow visualization and real-time, microscopic, volumetric rendering.

**Conclusions:**

Our study demonstrates the advantage of diffraction-based multifocal imaging techniques for 3D imaging of mm-scale objects from a single-camera exposure, with important applications in functional neural imaging and other areas benefiting from volumetric imaging.

## 1 Introduction

Imaging is inherently a two-dimensional (2D) process; however, many biological samples of interest are three-dimensional (3D). This presents a challenge for traditional microscopy when fast, dynamic processes such as neuronal activity are the object of study. With these methods, volumetric imaging rates are hampered by the relatively slow movement of high inertia focusing mechanisms.^1^^–^^3^ Recent technological advances have improved the speed of these focal plane shifts through rapid movement of the objective using piezo drives, “remotely” focusing the microscope with low-inertia devices or completely inertia-less defocusing methods.^4^^,^^5^ Still, volumetric rates over relevant tissue volumes remain inadequate for most fast dynamical processes.^5^ Together with the need for imaging large numbers of neurons over large fields of view (FOV), dynamic volumetric imaging is a challenge that is not fully met using these “serial” acquisition methods.

To further advance volumetric imaging rates, other recent light sheet^6^^–^^8^ and light field-based methods leverage the incredible advances in imaging sensor technology for large-scale parallelism in the spatial domain and fast acquisition speeds. Light field microscopy (LFM)^9^^,^^10^ is one such spatially parallelized volumetric imaging technique that enables volumetric imaging of fluorescent and non-fluorescent samples. By measuring the light pattern at the focal plane of a lenslet array, both spatial and angular information about the object are gathered, and 3D information can be reconstructed.^10^ However, because the quality of the volumetric reconstruction depends on the number of lenslets, there is a fundamental trade-off between resolution and depth: As fewer camera pixels are capturing light from the object at a specific angular position when the lenslets are small, the spatial resolution of the LFM image is compromised relative to conventional 2D imaging and is not uniform along all depths.^10^ Although benefiting from high-resolution camera sensors, LFM does not optimally use the high pixel counts of modern sCMOS sensors, and the time-consuming and computation-intensive reconstruction process^10^^–^^12^ severely underutilizes real-time visualization capabilities theoretically afforded by high frame rate cameras.

Multifocus microscopy (MFM) techniques offer a possible solution to these primary limitations of LFM. These methods reassign depth information from multiple focal planes into a tiled 2D array that can be imaged simultaneously on a camera.^13^ Sub-cellular resolution can be easily maintained even over large volumes by leveraging modern, large-format imaging sensors, in a more efficient manner than LFM, with speeds limited only by the camera frame rate. These techniques have been used for decades in various forms, including using beam-splitting strategies with single^14^^,^^15^ or multiple cameras,^15^^,^^16^ multiple imaging lenses of varied focal lengths,^17^ and diffractive optical elements (DOEs).^18^^–^^21^ These methods also have the advantage of directly acquiring adjacent focal planes without the need for computational reconstruction, enabling real-time volumetric visualization. The DOE approach in particular is promising due to its simplicity, scalability to many simultaneous focal planes, and long history of use.^13^ Nevertheless, these methods are typically used to image tiny volumes for particle tracking applications.^18^^–^^21^ Although they have been demonstrated for fluorescence functional neuroimaging^19^ of a small volume (40×40×16  μm), they have not found widespread use in large FOV volumetric fluorescence imaging typically used in systems neuroscience.^5^

**Fig. 1:**
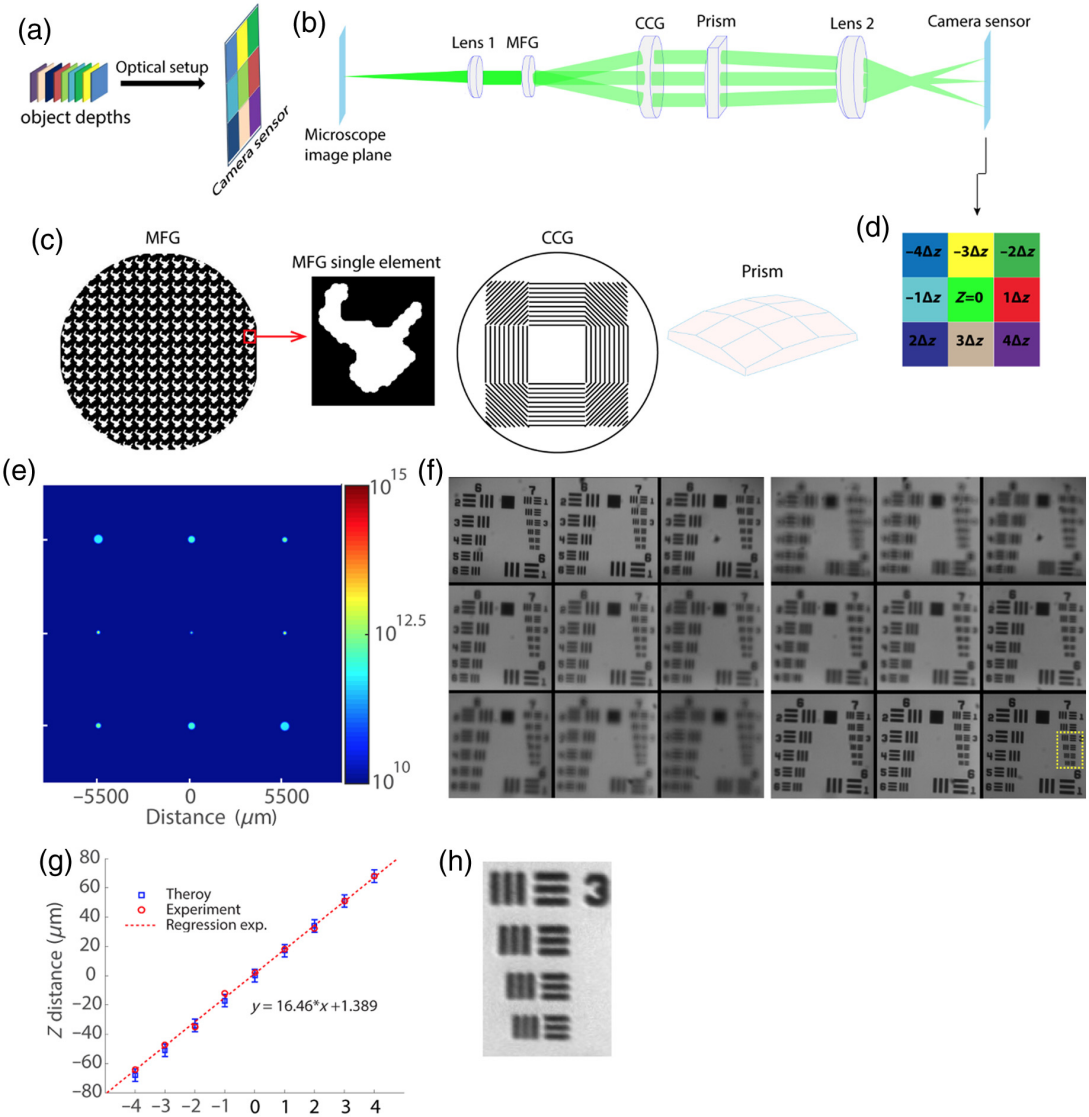
MFG-based multifocal imaging. (a) Imaging concept. 3D imaging is achieved by transforming the different focal plane images into a mosaic of images on the camera sensor. (b) Schematic of the optical design. The multiple depths optical imaging setup is based on three customized optical elements. The first element (MFG) is a diffraction grating that is responsible for splitting the microscope image into N×N different images (diffraction orders) while adding to each order a phase curvature to refocus different focal depths. The CCG and prism are responsible for correcting the chromatic dispersion caused by MFG. (c) Zoomed view of the different customized elements described in panel (b). Both CCG and prism are made of nine different panels, each corresponding to a different direction of the MFG diffraction light. The central panel is a plain optical window. (d) The mosaic of images on the camera sensor for the case of nine focal planes, with their associated diffraction orders from the grating. (e) Simulation validation of the nine PSFs obtained by a single point source at the focal plane. (f) Experimental validation of the method for the 10× microscope objective. The two extreme countercases of defocus are shown. (g) The stage shift distance versus each sub-image best focus. The experimental depth separation (graph slope, 16.46  μm) corresponds well to the original design (17  μm) and is well within the error bars range (the objective depth of field). (h) Zoom of the USAF resolution target verifying that the spatial resolution was not reduced, allowing for a resolution better than 2  μm/cycle.

Here, we present a powerful adaptation of the multifocal DOE approach for real-time acquisition of a large number of focal planes simultaneously, with diffraction-limited spatial resolution and importantly, over large (mm scale) volumetric FOVs. Similar to previous work,^18^ our technique achromatically diverts multiple focal plane images into different offset positions on the camera sensor, so they can be acquired in a single, fast (up to 10 ms) camera exposure [Fig. 1(a)]. The uniqueness of this work is its extension to low magnification and low NA configurations, enabling the extension of MFM to the rapid, 3D functional imaging of neural activity across FOVs containing hundreds of neurons. The novel versatility in our implementation also enables other important applications such as volumetric visualization of single blood cell flow in Zebrafish larvae and one-shot rendering of microscopic 3D objects.

## 2 Materials and Methods

Similar to earlier work in this field, our multifocal system is added to a Nikon TE2000-U inverted microscope and consists of three customized elements that form N×N different images on the camera sensor (Andor Zyla 4.2 sCMOS), each of a different depth in the sample. The first element, a multifocal grating (MFG), is a 2D diffractive grating that has two roles: splitting the light into N×N different orders and adding an inverse phase curvature to each order to compensate for the defocus of each object’s depth. The splitting of light into $N^{2}$ directions is achieved with a binary phase grating that was designed by a pixel flip phase retrieval algorithm adapted from previous work^18^ for an object of N×N point objects. To obtain large FOV images at each depth, we designed our element to have nine different sub-images (N=3) with a light efficiency of 60% after optimizing for multifocal performance factors such as the focal plane spacing and uniform brightness across sub-images [Fig. 1(b)]. This gives three orders of diffraction in both the x and y axes ($m_{x}$, $m_{y}=0$, ±1). The inverse defocus phase compensation to each order of diffraction was done by a geometrical distortion at each axis of the grating^13^ according to

$$\delta x(x_{p},y_{p})=(d/\lambda )\times n\times \Delta z\times \sqrt{1-(x_{p}^{2}+y_{p}^{2})/(n\times f_{\text{obj}}{)}^{2}},\;\delta y(x_{p},y_{p})=N\times \delta x(x_{p},y_{p}),$$ (1)

where ($x_{p},y_{p}$) is the objective pupil plane coordinates, d is the grating period, n is the refractive index of the medium between the sample and the objective, λ is the wavelength, Δz is the object depth separation, and $f_{\text{obj}}$ is the focal length of the microscope objective. Applying this kind of distortion on the grating will form a $\Delta z\times (m_{x}+N\times m_{y})$ focus shift to each $\left(m_{x},m_{y}\right)$ diffraction order. Consequently, the MFG forms a matrix of images on the camera sensor, each of different focal depths [Fig. 1(d)]. The MFG is relayed to the objective pupil plane to create a conjugate image of the object at the camera [Fig. 1(b)].

MFGs serve as an excellent solution for the case of multiple-depth imaging of monochromatic light. However, for polychromatic light, such as fluorescence emission from common fluorophores, the linear dependence of the wavelength on the diffraction angle in a diffraction grating^22^ will form chromatic dispersion that distorts the image. To overcome this problem, we designed a chromatic correction grating (CCG) consisting of a customized blazed grating and prism. The MFG and CCG were custom-manufactured by Holo/Or. The blazed grating is made of N×N different panels, each designed to compensate for the chromatic dispersion of an individual MFG order of diffraction. This is done by deflecting the angle of diffraction of each MFG order (for the central wavelength) by its inverse angle. Correcting the chromatic dispersion of the MFG inverts the angular separation of the focal planes, leading to overlapping images at the camera sensor. To compensate for this effect, a customized prism was added to deflect each order back to the inverse angular direction that was formed by the MFG.^18^ Both the chromatic correction grating and the prism are made of $N^{2}$ panels, each oriented according to the expected diffraction angle from the MFG (the central panel that corresponds to light that was not diffracted by the MFG is a plane window). Figure 1(c) shows the drawing of each of the customized elements.

Unlike previous multifocal microscopes that were aimed at high magnification via a specific high-power objective, our goal was to image large numbers of depths at large FOVs with the flexibility to use different objectives with various magnifications. The condition of using different objectives with the same setup is found by calculating the MFG size:

$$D_{\text{MFG}}=M_{\text{obj}}\cdot D_{\text{obj}}=\frac{f_{\text{tube}}}{f_{1}}\times (2N.A\times f_{\text{obj}}),$$ (2)

where $M_{\text{obj}}$ is the objective magnification, $D_{\text{obj}}$ is the pupil size diameter, $f_{\text{tube}}$ is the microscope tube lens focal length, $f_{1}$ is the first relay lens focal length, and N.A is the objective numerical aperture (NA). Because the microscope tube lens and relay lens focal length are well known, Eq. (2) is written as a linear equation of the NA and objective magnification:

$$D_{\text{MFG}}=2cN.A\times f_{\text{obj}}=2c\text{N}.\text{A}\times \frac{f_{\text{tube}}}{M_{\text{obj}}},$$ (3)

where c is a constant factor equal to $f_{\text{tube}}/f_{1}$. By taking into account that $f_{\text{tube}}$ is also constant, using different objectives is possible as long as the ratio of $N.A/M_{\text{obj}}$ does not change. In our design, we used a 10×, N.A 0.25, and a 4×, N.A 0.1 objective (Olympus). Higher NA objectives that do not adhere to the $N.A/M_{\text{obj}}$ constraint could be utilized, but this would result in an altered pupil size, and a redesigned MFG would be required. Alternatively, demagnification of the pupil’s additional optics could be used, at the expense of a change in the design focal spacing. For the 10× objective, the focal depth separation between planes was designed to be 17  μm, giving a total depth of field of 8×17=136  μm, whereas for the 4× objective, the depth separation was restricted by Eq. (1) to be 115  μm (total depth of 920  μm). To avoid overlapping between different depths, the depth separation was chosen to be well above the depth of field of the objectives. The FOV of each image on the sensor is $0.5\times 0.5\;{\mathrm{mm}}^{2}$ and is $1.2\times 1.2\;{\mathrm{mm}}^{2}$ for the different microscope objectives. The chromatic correction of our setup enables a bandwidth of 520±20  nm, which suits various genetically encoded fluorescent indicators (GCaMPs) as well as bright field imaging applications.

## 3 Results

We first validated the physical characteristics of our method with a series of optical simulations. We verified the design of our MFG by simulating nine different point sources at the depths that correspond to the desired depth corrections. The obtained point spread function at each depth demonstrates the depth correction [Fig. 1(e)]. The validation of the chromatic correction as well as the agreement with the camera sensor dimensions (to avoid overlapping between the sub-images) was done by ray transfer matrix analysis simulation, which gave us both the transverse and angular positions of each ray at each element position (see Fig. S1 in the Supplementary Material for the chromatic correction validation). To experimentally validate our multiple-depth ability, we focused each of the sub-images using a 2D USAF resolution target as an object and gradually taking the microscope out of focus [Fig. 1(f)]. By measuring the vertical shift of the microscope stage from its focus plane, we can measure the focal depth separation between the planes of each sub-image. This analysis shows excellent agreement with our design [Fig. 1(g)], as well as the method’s excellent spatial resolution [Fig. 1(h)].

**Fig. 2:**
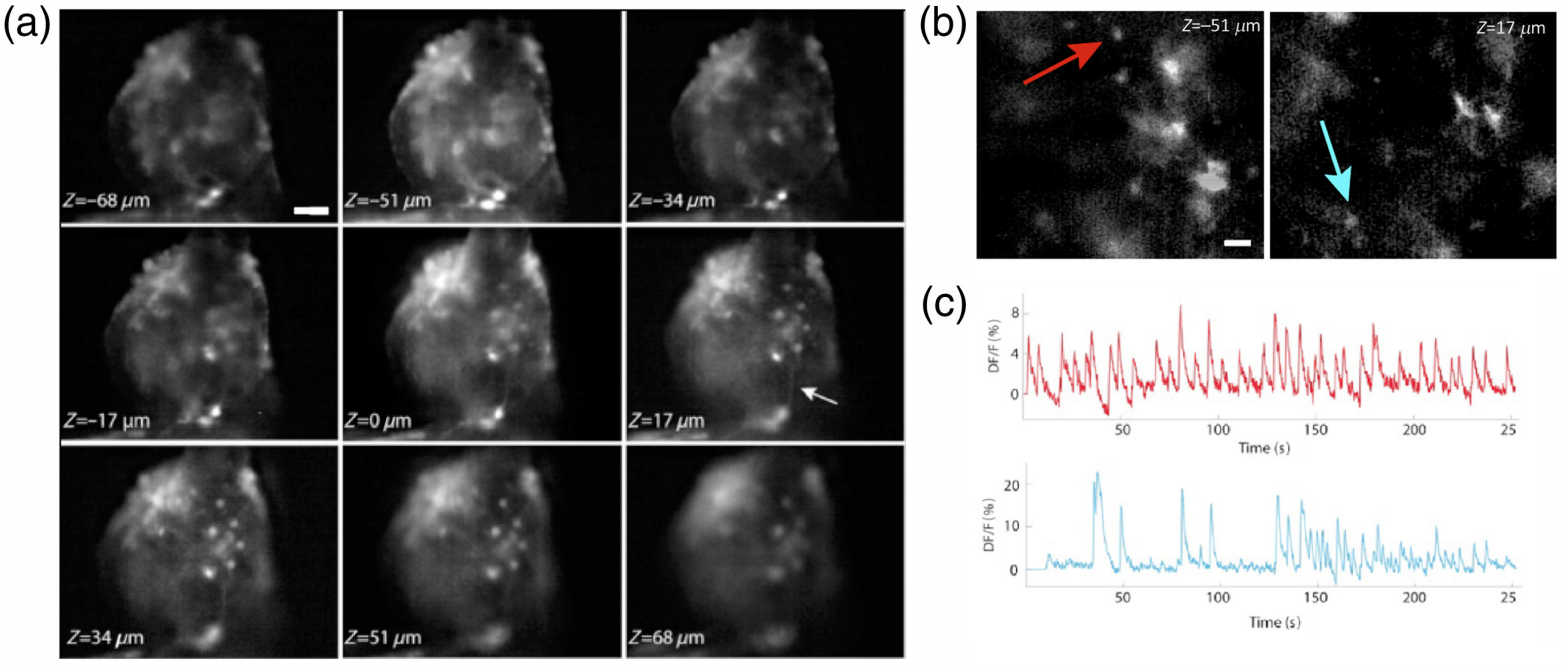
Experimental results for the neural Optonet culture. (a) The different depths images of the Optonet as taken by a single image of the multiple-depth microscope. The different cells in focus at different layers demonstrate the optical sectioning of this method. Notice the axon in panel 6 that connects two cells at different depths (denoted by an arrow). Scale bar=50  μm. (b) An example of two different depth images that were captured simultaneously for measurement of fast neural activity. (c) Spontaneous neural activity of two different cells [denoted by corresponding colors in panel (b)]. The results show correlated and independent activity of the cells. Scale bar=30  μm.

To test our system’s ability to visualize large-scale neural networks, we first imaged 3D neural “Optonet” cultures,^23^^–^^25^ an optically accessible and sensitive bioengineered cortical neural network exhibiting spontaneous neural activity. Imaging of these samples was done using a 10× objective at a 5 Hz frame rate. To further improve the in-plane resolution, we deconvolved each of the frames with the compatible 2D PSF, which was measured in advance with a sub-diffraction-limit bead.^26^ The application of the multiple-depth technique to the Optonet cultures enables the observation of different cells over the entire volume while preserving effective resolution, further allowing for the visualization of axons between cells at different depths [Fig. 2(a)]. We also measured the spontaneous neural activity of an Optonet culture that expresses the genetically encoded Ca^2+^ indicators (GECI) GCaMP6m [Fig. 2(b)], demonstrating that sub-cellularly resolved, independent neural activity can be observed throughout a large volume. Figure 2(c) shows the spontaneous activity of two cells in the volume, having both synchronized and independent ${\mathrm{Ca}}^{2+}$ traces.

**Fig. 3:**
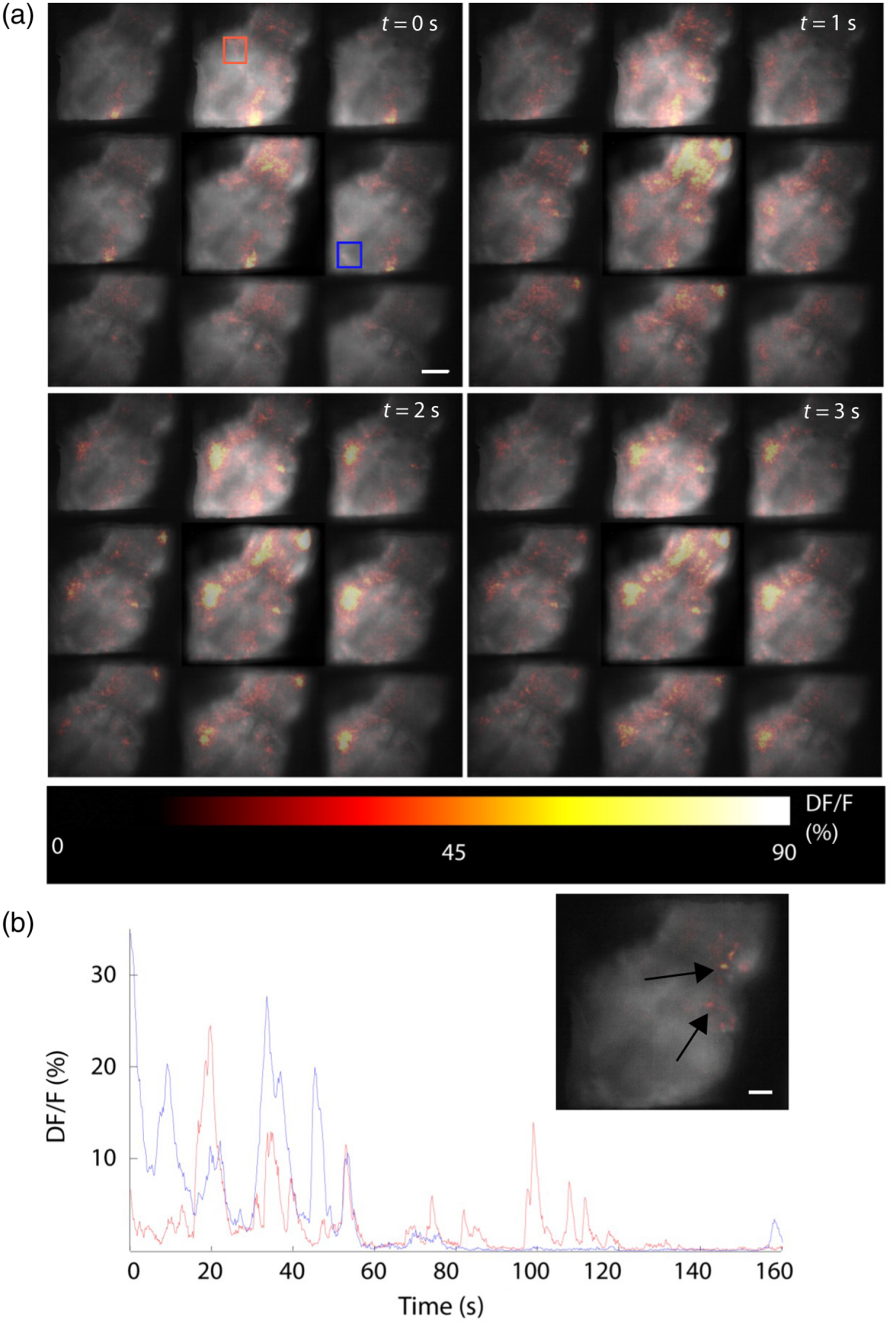
Simultaneous multiple depths calcium imaging *in vivo*. (a) Demonstration of the neural activity imaging at different depths of the larval zebrafish. Spatially and temporally distributed neural activity is shown across the midbrain and part of the forebrain with single-cell resolution. Scale bar: 100  μm, Δz=17  μm. (b) Neural activity of two single subregions [denoted by colored rectangles in panel (b)], demonstrating both independent and correlated neural activity. Inset, Image showing putative single-cell-resolved functional activity. Scale bar: 50  μm.

We next used our multifocal strategy in Tg(HuC:GCaMP5G)^27^ Zebrafish larvae expressing GCaMP5G pan-neuronally, in which network hyper-activity was induced by the application of 0.25 mM 4-AP, a potassium channel inhibitor. The larval zebrafish is a popular model for linking large-scale neural activity to simple behaviors. To better differentiate the neural activity from the background image, we subtracted the average image from each of the frames and visualized the ${\mathrm{Ca}}^{2+}$ events by pseudocolor [Fig. 3(a) and Video 1]. To further reduce the crosstalk effect of the scattered light across depths, we weakly thresholded the activity pattern (passing 95% of the detected signal at each depth, after first applying Gaussian spatial smoothing). The obtained neuronal activity pattern demonstrates peaks at different sub-regions in different planes as well as the time progress of the network along the volume at a resolution of single-cell activations [Figs. 3(a)–3(d) and Video 1]. Figure 3(b) shows the activity of two different areas inside the volume, demonstrating both synchronized and non-synchronized activity in those areas.

**Fig. 4:**
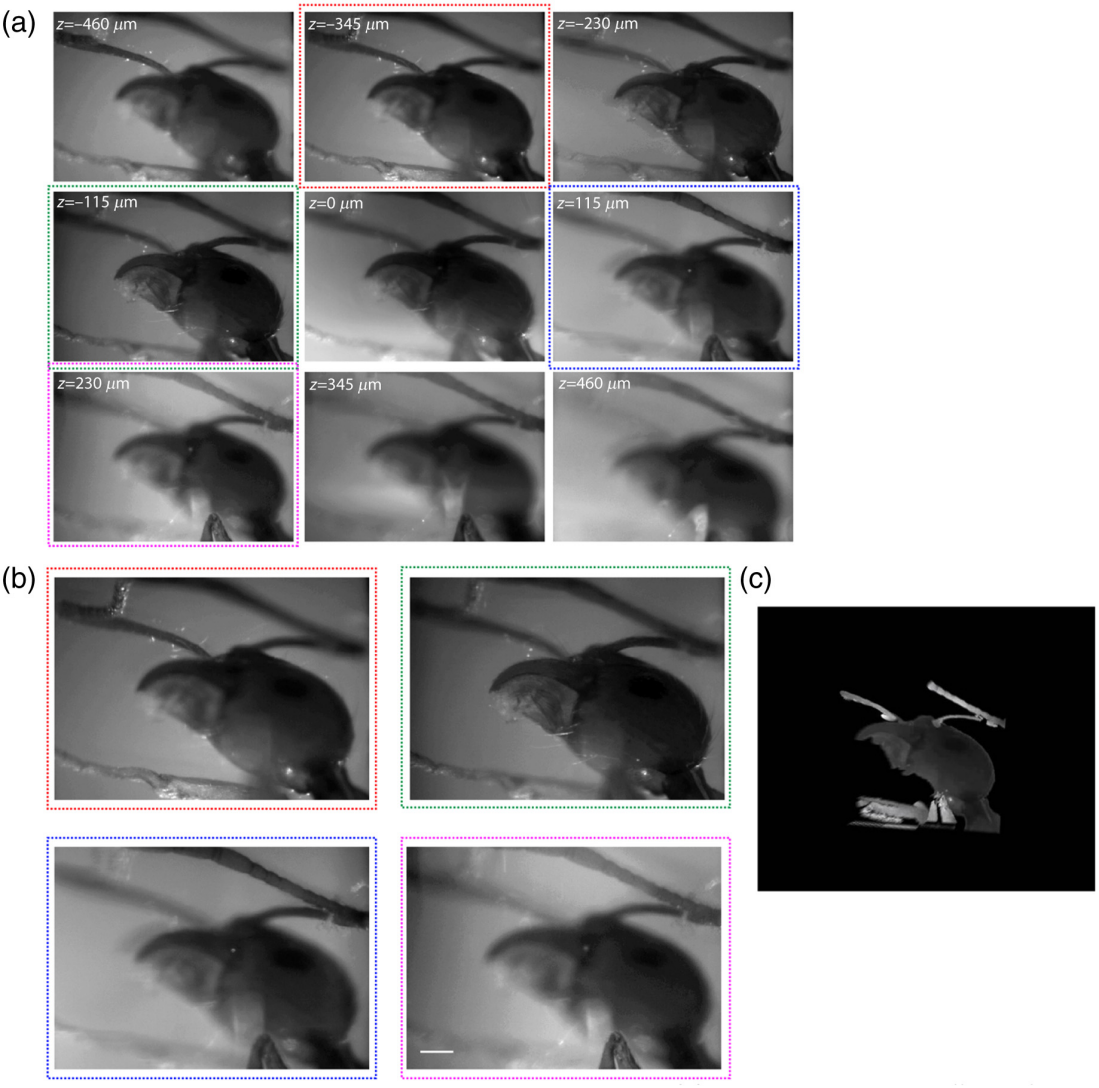
3D reconstruction by a single multiple-depth image. (a) An ant head at nine different focal planes, taken by a single camera exposure. (b) Zoomed view of four different sub-images showing how different parts of the ant head are in focus at different depths. Scale bar, 75  μm. (c) 3D reconstruction of the head after removal of the out-of-focus data from (b).

In addition to its application in multiple-depth visualizations of neural networks and their activity, the fast acquisition rate of this technique, limited only by the camera’s frame rate, can potentially be used in other interesting applications, such as visualizing 3D blood flow. To test this possibility, we imaged an agarose-embedded Zebrafish larva’s tail at 100 frames per second (camera readout limit), using brightfield imaging with the 4× objective. We confirmed that this enabled us to discern the flow of single blood cells at different depths, including the observation of high-velocity flow rates in veins as well as slower ones in capillaries (Video 2) at almost 1 mm depth. Finally, we also used our technique to surface-render a continuous 3D outline of a mesoscopic object from the nine independent focal depths. This was done by removing the out-of-focus information of each depth by a semi-automatic image processing algorithm that seeks sharp edges to estimate the focused region. For instance, we demonstrate this for an ant head [Figs. 4(a) and 4(b), see also Video 3]. The 3D rendering was done using a commercial software package (Imaris, Oxford Instruments, UK).

## 4 Discussion and Conclusion

In this study, we demonstrated a technique that enables rapid imaging of large volumes at microscopic resolution, using both fluorescence and bright-field modalities. Inspired by previous work on diffractive multi-focus microscopes,^18^^–^^21^ the main advance in this work is the transition to the low magnification/large FOV domain, which now enables covering large areas (millimeter-scale) in each sub-image. This development moves multifocal techniques away from the small FOV, high NA, particle tracking regime into the realm of biological systems and large-scale functional neurophotonics, both of which benefit from volumetric imaging. This application domain requires both high spatial resolution and a large FOV, in addition to excellent temporal resolution. As an additional improvement, we also show the versatility of this approach to different microscope objectives by setting a selection rule for the objectives and demonstrating our approach with two different magnifications. These capabilities are becoming increasingly desirable in neuroscience to take advantage of next-generation GECIs and voltage indicators,^28^^,^^29^ which require extremely high volumetric rates to accurately sample.

In comparison with LFM, which also enables scan-less volumetric imaging, at the price of a lower spatial resolution, the trade-off in our approach is the limited field of view rather than the resolution. However, this limitation is generally much easier to overcome as it only requires a larger imaging sensor. In addition, this multiple-depth technique has other distinct advantages: the extraction of the volume information is immediate, all depths have the same resolution (which is the microscope resolution), there is no need for prior calibration of the setup, and it is simple to use. At the theoretical level, one of the major advantages of our method over the microscopic light field is that the point spread function is space invariant, as in most imaging techniques. This allows us to use conventional 3D deconvolution algorithms to reduce the out-of-focus crosstalk between the different planes. In future studies, we intend to investigate different algorithms for this goal.^30^ Although our method in its current implementation is most suitable for transparent or semi-transparent samples, in many cases, the effect of out-of-focus crosstalk can be reduced computationally (as described) or optically by designing a setup with depth separation that is much larger than the depth of field of the microscope objective. Moreover, further development of near-infrared functional indicators will further mitigate these issues,^31^ especially in shallow neocortical structures.

In addition to the important application of this approach in the field of neuroscience,^29^ the ability to measure large volumes rapidly without degradation of resolution has promising applications in the study of fluidics (see Video 2). It can also be advantageous in essentially all cases in which scan-based microscopic techniques are not fast enough to capture a dynamic process in all three dimensions. Moreover, the simplicity of this method, which enables scanless 3D microscopic imaging at a single camera exposure, may promote it as a favorable alternative even in cases in which high temporal resolution is not essential (e.g., Fig. 4).

## 5 Appendix: Supplemental Videos

**Video 1.** Multifocal functional fluorescence imaging in an agarose-embedded Zebrafish larvae (Fig. 3). The neurons express the calcium indicator GCaMP5G, and network hyper-activity was induced pharmacologically (Video 1, MP4, 6.08 MB [URL: https://doi.org/10.1117/1.NPh.11.S1.S11515.s1]).
**Video 2.** Multifocal blood-flow imaging in a Zebrafish larva’s tail at 100 frames per second. Blood cells flowing at different depths and at different speeds can be visualized (Video 2, MP4, 4.01 MB [URL: https://doi.org/10.1117/1.NPh.11.S1.S11515.s2]).
**Video 3.** 3D surface rendering of an ant’s head. The object’s continuous outline was extracted from the nine independent focal depths in Fig. 4 (Video 3, MP4, 2.48 MB [URL: https://doi.org/10.1117/1.NPh.11.S1.S11515.s3]).

## Supplementary Material









## Data Availability

The data that support the findings of this study are available from the corresponding author upon reasonable request.

## References

[1] Denk W. Strickler J. H. Webb W. W., “Two-photon laser scanning fluorescence microscopy,” Science 248, 73–76 (1990). SCIEAS 0036-8075 10.1126/science.2321027 2321027

[r2] Keller P. J. et al., “Reconstruction of zebrafish early embryonic development by scanned light sheet microscopy,” Science 322, 1065–1069 (2008). SCIEAS 0036-8075 10.1126/science.1162493 18845710

[3] Paddock S. W., “Confocal laser scanning microscopy,” Biotechniques 27, 992–1004 (1999). BTNQDO 0736-6205 10.2144/99275ov01 10572648

[4] Lecoq J. Orlova N. Grewe B. F., “Wide, fast, deep: recent advances in multiphoton microscopy of in vivo neuronal activity,” J. Neurosci. 39, 9042–9052 (2019). JNRSDS 0270-6474 10.1523/JNEUROSCI.1527-18.2019 31578235 PMC6855689

[5] Wu J. Ji N. Tsia K. K., “Speed scaling in multiphoton fluorescence microscopy,” Nat. Photonics 15, 800–812 (2021). NPAHBY 1749-4885 10.1038/s41566-021-00881-0

[6] Olarte O. E. et al., “Decoupled illumination detection in light sheet microscopy for fast volumetric imaging,” Optica 2, 702–705 (2015). 10.1364/OPTICA.2.000702

[r7] Quirin S. et al., “Calcium imaging of neural circuits with extended depth-of-field light-sheet microscopy,” Opt. Lett. 41, 855–858 (2016). OPLEDP 0146-9592 10.1364/OL.41.000855 26974063 PMC4894304

[8] Tomer R. et al., “SPED light sheet microscopy: fast mapping of biological system structure and function,” Cell 163, 1796–1806 (2015). CELLB5 0092-8674 10.1016/j.cell.2015.11.061 26687363 PMC4775738

[9] Nöbauer T. Vaziri A., Light field microscopy for in vivo Ca^2+^ imaging, Handbook of Neurophotonics, Pavone F. S. Shoham S., Eds., CRC Press (2020).

[10] Broxton M. et al., “Wave optics theory and 3-D deconvolution for the light field microscope,” Opt. Express 21, 25418–25439 (2013). OPEXFF 1094-4087 10.1364/OE.21.025418 24150383 PMC3867103

[r11] Nöbauer T. et al., “Video rate volumetric Ca^2+^ imaging across cortex using seeded iterative demixing (SID) microscopy,” Nat. Methods 14, 811–818 (2017).1548-7091 10.1038/nmeth.4341 28650477

[12] Prevedel R. et al., “Simultaneous whole-animal 3D imaging of neuronal activity using light-field microscopy,” Nat. Methods 11, 727–730 (2014).1548-7091 10.1038/nmeth.2964 24836920 PMC4100252

[13] Blanchard P. M. Greenaway A. H., “Simultaneous multiplane imaging with a distorted diffraction grating,” Appl. Opt. 38, 6692–6699 (1999). APOPAI 0003-6935 10.1364/AO.38.006692 18324206

[14] Xiao S. et al., “High-contrast multifocus microscopy with a single camera and z-splitter prism,” Optica 7, 1477–1486 (2020). 10.1364/OPTICA.404678 34532564 PMC8443084

[15] Geissbuehler S. et al., “Live-cell multiplane three-dimensional super-resolution optical fluctuation imaging,” Nat. Commun. 5, 5830 (2014). NCAOBW 2041-1723 10.1038/ncomms6830 25518894 PMC4284648

[16] Sacconi L. et al., “KHz-rate volumetric voltage imaging of the whole zebrafish heart,” Biophys. Rep. 2, 100046 (2022). 10.1016/j.bpr.2022.100046 PMC9680780 36425080

[17] Hansen J. N. et al., “Multifocal imaging for precise, label-free tracking of fast biological processes in 3D,” Nat. Commun. 12, 4574 (2021). NCAOBW 2041-1723 10.1038/s41467-021-24768-4 34321468 PMC8319204

[18] Abrahamsson S. et al., “Fast multicolor 3D imaging using aberration-corrected multifocus microscopy,” Nat. Methods 10, 60–63 (2013).1548-7091 10.1038/nmeth.2277 23223154 PMC4161287

[r19] Abrahamsson S. et al., “Multifocus microscopy with precise color multi-phase diffractive optics applied in functional neuronal imaging,” Biomed. Opt. Express 7, 855–869 (2016). BOEICL 2156-7085 10.1364/BOE.7.000855 27231594 PMC4866461

[r20] Dalgarno P. A. et al., “Multiplane imaging and three dimensional nanoscale particle tracking in biological microscopy,” Opt. Express 18, 877–884 (2010). OPEXFF 1094-4087 10.1364/OE.18.000877 20173908

[21] Hajj B. et al., “Whole-cell, multicolor superresolution imaging using volumetric multifocus microscopy,” Proc. Natl. Acad. Sci. U. S. A. 111, 17480–17485 (2014). 10.1073/pnas.1412396111 25422417 PMC4267334

[22] Lipson A. Lipson S. G. Lipson H., Optical Physics, Cambridge University Press (2010).

[23] Dana H. et al., “Hybrid multiphoton volumetric functional imaging of large-scale bioengineered neuronal networks,” Nat. Commun. 5, 3997 (2014). NCAOBW 2041-1723 10.1038/ncomms4997 24898000 PMC4113029

[r24] Marom A. et al., “Microfluidic chip for site-specific neuropharmacological treatment and activity probing of 3D neuronal “Optonet” cultures,” Adv. Healthcare Mater. 4, 1478–1483 (2015). 10.1002/adhm.201400643 25953011

[25] Marom A. et al., “Spontaneous activity characteristics of 3D “optonets”,” Front. Neurosci. 10, 602 (2017).1662-453X 10.3389/fnins.2016.00602 28119555 PMC5220075

[26] Lucy L. B., “An iterative technique for the rectification of observed distributions,” Astron. J. 79, 745 (1974). ANJOAA 0004-6256 10.1086/111605

[27] Pérez-Schuster V. et al., “Sustained rhythmic brain activity underlies visual motion perception in zebrafish,” Cell Rep. 17, 1098–1112 (2016). 10.1016/j.celrep.2016.09.065 27760314 PMC5081404

[28] Abdelfattah A. S. et al., “Neurophotonic tools for microscopic measurements and manipulation: status report,” Neurophotonics 9, 013001 (2022). 10.1117/1.NPh.9.S1.013001 35493335 PMC9047450

[29] Zhang Y. et al., “Fast and sensitive GCaMP calcium indicators for imaging neural populations,” Nature 615, 884–891 (2023). 10.1038/s41586-023-05828-9 36922596 PMC10060165

[30] Sarder P. Nehorai A., “Deconvolution methods for 3-D fluorescence microscopy images,” IEEE Signal Process Mag. 23, 32–45 (2006). ISPRE6 1053-5888 10.1109/MSP.2006.1628876

[31] Qian Y. et al., “A genetically encoded near-infrared fluorescent calcium ion indicator,” Nat. Methods 16, 171–174 (2019).1548-7091 10.1038/s41592-018-0294-6 30664778 PMC6393164

