# Supplementary material for: Multifocal microscopy for functional imaging of neural systems
Source: Neurophotonics. 2024 Sep 17;11(Suppl 1):S11515. doi: 10.1117/1.NPh.11.S1.S11515 (PMC11407684; doi:10.1117/1.NPh.11.S1.S11515)
Supplement: Supplementary file 1 [file NPh_011_S11515_SD001.pdf]

Supplementary information

Chromatic correction validation

To validate that there is no chromatic deviation for different wavelengths that are within our bandwidth and to make sure that the subimages are not overlapping, we ran a ray tracing simulation that was based on ray transfer matrix analysis. Figure S1 show the results for two extreme counter wavelengths which that without any correction will yield the larger deviation. The result of this simulation validate that for both wavelengths the deviation is much smaller than the size of the camera pixel. The distance between the orders is 5.5 [mm] which is 1/3 of the camera sensor size.

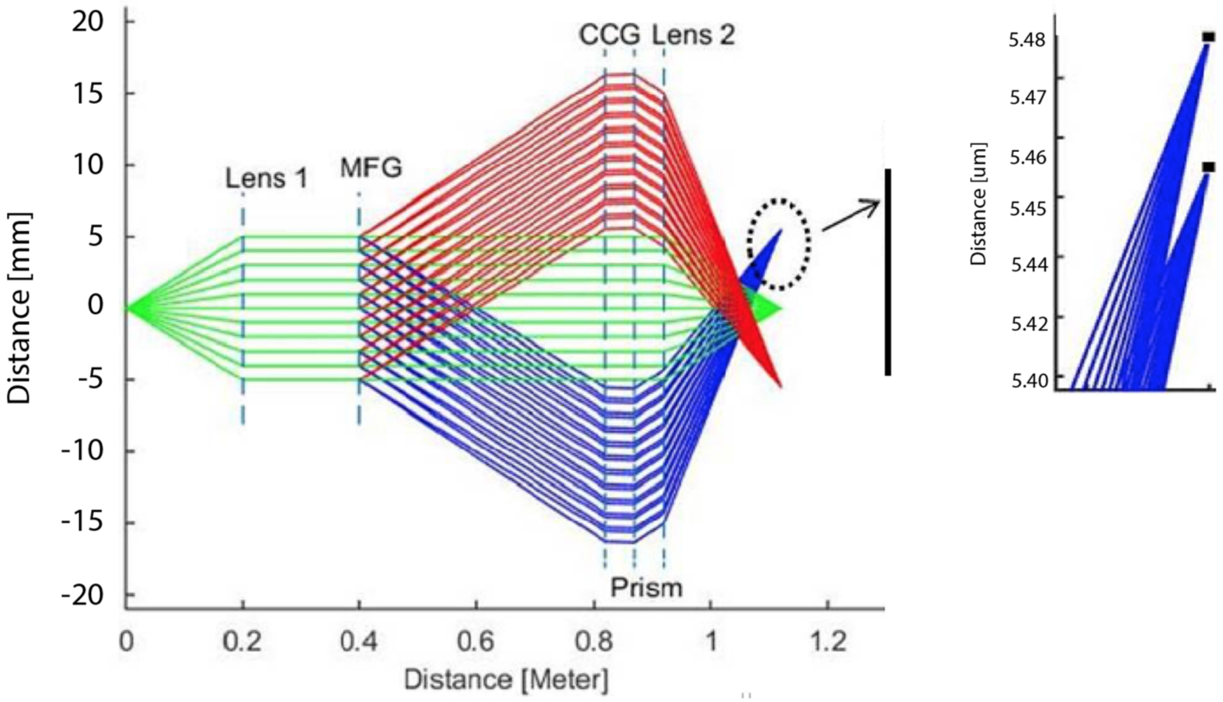

Figure S1. Chromatic deviation correction. Left panel shows a ray tracing simulation, for the two extreme counter wavelengths in our bandwidth (λ=502, 535 nm) of the path and angular separation of the different focal planes as produced by the MFG. To avoid overlapping between the sub-images the distance between the orders is 5.5 mm which is 1/3 of camera sensor size (bar). Right, the chromatic distortion/displacement between the extremal spectral points. The chromatic deviation is very small compared to the pixel size of the camera (0.02 μm with respect to 6.5 μm).
